# Enzyme-instructed morphology transformation of mitochondria-targeting peptide for the selective eradication of osteosarcoma†

**DOI:** 10.1039/d2cb00166g

**Published:** 2022-10-17

**Authors:** M. T. Jeena, Seongeon Jin, Batakrishna Jana, Ja-Hyoung Ryu

**Affiliations:** Department of Chemistry, Ulsan National Institute of Science and Technology (UNIST) Ulsan 44919 Republic of Korea jhryu@unist.ac.kr

## Abstract

The treatment of osteosarcoma involves an adjuvant therapy that combines surgery and chemotherapy. However, considering that children are the main victims of osteosarcoma, replacing such a harsh treatment with a soft but powerful method that ensures a complete cure while having no adverse effects is highly desirable. To achieve this aim, we have developed a supramolecular therapeutic strategy based on morphology-transformable mitochondria-targeting peptides for the eradication of osteosarcoma with enhanced selectivity and reduced side effects. A newly designed micelle-forming amphiphilic peptide, l-Mito-FFYp, consisting of a phosphate substrate for the biomarker enzyme of osteosarcoma alkaline phosphatase (ALP), disassembles in response to the ALP enzyme in the cell membrane to generate positively charged l-Mito-FFY molecules, which diffuse inside the targeted cell and self-assemble to form nanostructures specifically inside the mitochondria to induce cell apoptosis.

## Abstract 2

Enzyme instructed morphology transformation for selective cancer therapy. Due to the response of alkaline phosphatase, micelle was disassembled into a monomer, it internalized to the cell and induced mitochondrial dysfunction through self-assembly.

## Graphical abstract

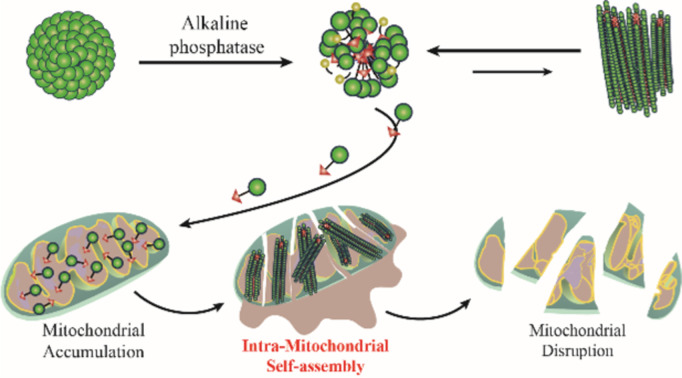

## Introduction

Osteosarcoma is a pleomorphic malignant bone tumor that commonly develops in the femur, tibia, and humerus^1^ and generally affects children and adults of age between 10 and 20. This type of cancer has high propensity for metastasis to lungs and sometimes to other bones,^2^ leading to a five-year survival rate for patients with metastatic osteosarcoma of only 10–20% despite the recent advances in chemotherapy.^3^ Osteosarcoma is often treated by combining chemotherapy with surgery or radiation.^4^ However, children are particularly sensitive to these methods and require milder therapies. Moreover, the chemotherapy used for the treatment of osteosarcoma is based on cytotoxic drugs such as doxorubicin, *cis*-platin, and high-dose methotrexate with leucovorin rescue.^5^ These drugs are toxic to both cancer and normal cells and often elicit severe side effects; thus, their administration to children is not recommended.^2,6^ Therefore, the development of a mild and safe treatment for children with reduced adverse effects is an urgent need.^7^ Recently, mitochondria-targeting supramolecular therapeutics have attracted the attention of researchers engaged in cancer therapy due to their overwhelming advantages over conventional therapies, such as enhanced tumor healing efficiency, reduced side effects, and lack of acquired resistance.^8–11^ Accordingly, mitochondria targeting supra-molecular therapy can be envisaged as a promising method for the treatment of osteosarcoma.^12,13^

**Scheme 1:**
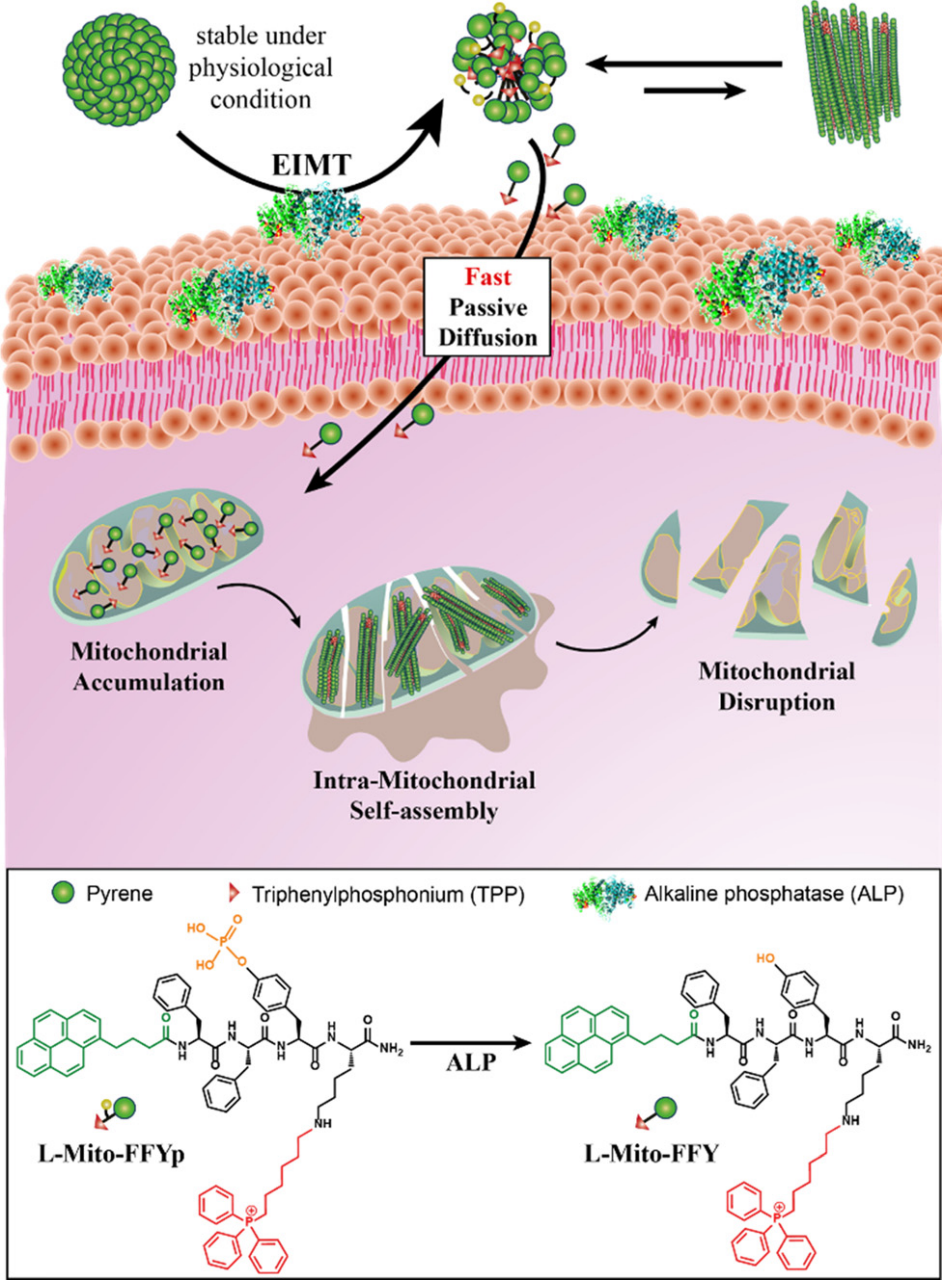
Schematic illustration of the enzyme-instructed morphology transformation (EIMT) of an ALP-responsive micelle. The ALP enzyme induces the transformation of l-Mito-FFYp micelles to positively charged Mito-FFY molecules, which enter the cell *via* free diffusion to target the mitochondria. Inside the mitochondria, l-Mito-FFYp self-assembles into nanofibers to induce mitochondrial dysfunction leading to cell death. Chemical structure of l-Mito-FFYp and l-Mito-FFY represented under the scheme.

In general, mitochondrion-targeting supramolecular therapy uses a nontoxic self-assembling small molecule (*e.g.*, an amphiphilic peptide or the monomer of a polymer) to target the tumor. The self-assembly of this agent within the mitochondria leads to cell death *via* mitochondrial disruption and the consequent release of apoptotic bodies.^14–19^ Promising *in vitro* and *in vivo* results have been demonstrated for this approach.^20,21^ However, the small molecules can be rapidly cleared from the body, and the positive charge demanded by mitochondrion-targeting molecules such as triphenylphosphonium (TPP)^22^ induces undesirable nonspecific interactions with negatively charged biomolecules and serum proteins, decreasing the tumor healing efficiency. In this study, this issue was addressed to develop a powerful strategy for osteosarcoma treatment. The self-assembling peptide Mito-FF (l-form),^23^ previously designed in our group, was modified to form micelles of mitochondrion-targeting supramolecular agents possessing negative outer surface charge. In addition, to achieve high selectivity, a l-tyrosine phosphate unit (Yp) was used as a substrate for the alkaline phosphatase enzyme (ALP),^24–26^ which is overexpressed in osteosarcoma cells,^27^ leading to the designed peptide l-Mito-FFYp. The binding between the phosphoric acid substrate and ALP inhibits the activity of the enzyme, which contributes to tumor growth suppression.^12,28,29^ ALP enzymes promote the tumor growth by producing immune suppressive adenosine.^30,31^ In response to ALP, the l-Mito-FFYp micelles disassemble to l-Mito-FFY molecules within the cell membrane. Due to the positive charge and hydrophobicity of the l-Mito-FFY molecules, they internalize in the cell to target the mitochondria prior to their self-assembly within the cell membrane. The molecules then self-assemble inside the mitochondria to induce mitochondrial dysfunction and cell death (Scheme 1). In this step, the place of self-assembly is confined to the mitochondria. Therefore, the amount of molecule required to reach the Critical Aggregation Concentration (CAC) which means the concentration at which self-assembly starts is small. It makes the anticancer effect can be enhanced even with a small amount.

## Results and discussion

### Design strategy and characterization of the enzyme-instructed morphology transformation (EIMT) of l-Mito-FFYp

**Fig. 1:**
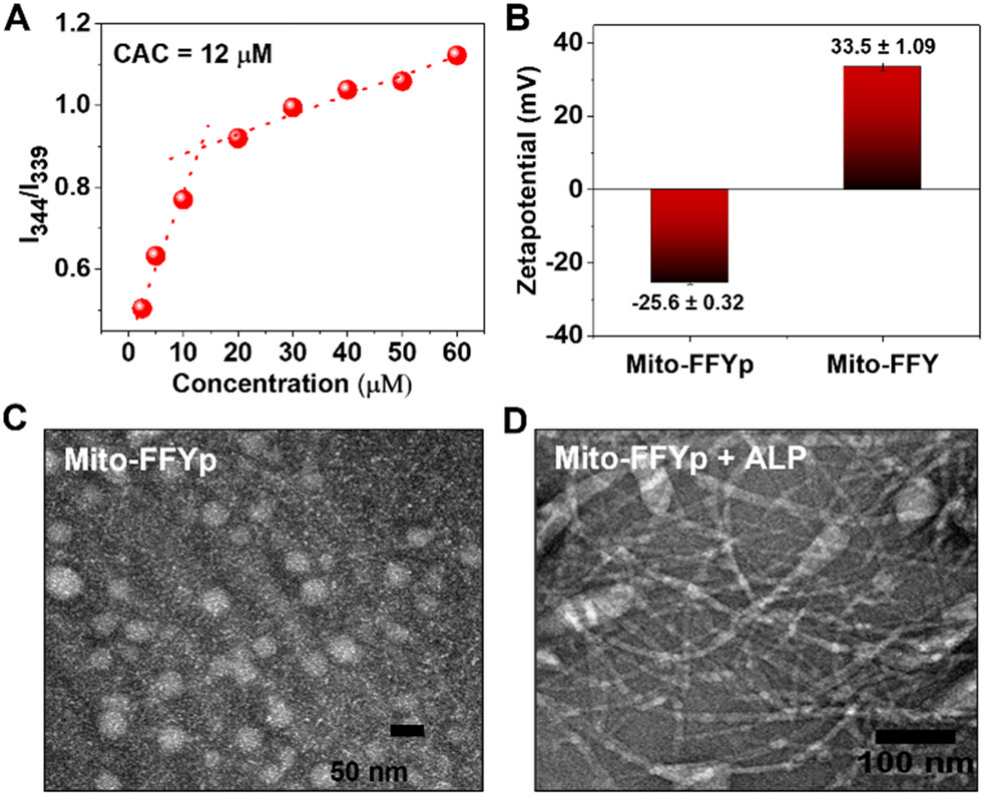
(A) CAC determination of l-Mito-FFYp using pyrene excitation method. (B) Surface charge analysis of l-Mito-FFYp and Mito-FFY showing negative potential for l-Mito-FFYp micelle and positive potential for Mito-FFY. TEM analysis of 15 μM of (C) l-Mito-FFYp and (D) l-Mito-FFYp + ALP.

l-Mito-FFYp consists of a tripeptide backbone Phe–Phe–Tyrp (Tyrp stands for tyrosine phosphate) in which pyrene butyric acid is capped on the N-terminus to promote the self-assembly and TPP is conjugated to the amine group of the lysine side chain for targeting the mitochondria (Fig. S1, ESI†). The CAC of l-Mito-FFYp was determined to be 12 μM *via* pyrene excitation method (Fig. 1A). According to a surface charge analysis, a 15 μM (over the CAC value) solution of l-Mito-FFYp showed a charge of −25.6 ± 0.32 mV, whereas that of Mito-FFY was +33.5 ± 1.09 mV, suggesting its easy cell internalization (Fig. 1B) due to the negative potential of cancer cell membrane.^32^ As shown in Fig. 1C, l-Mito-FFYp self-assembles into a micellar structure. The EIMT of l-Mito-FFYp was investigated by treating it with ALP enzyme. After 24 h incubation (37 °C), fibers having a diameter of *ca.* 10 nm were observed *via* transmission electron microscopy (TEM) analysis (Fig. 1D). Specifically, micelles with a diameter of 53 nm were converted into fibers with a diameter of 12.9 nm, most likely by the action of the ALP enzyme. To confirm that the fiber structure corresponded to l-Mito-FFY, l-Mito-FFY was separately synthesized and purified (Fig. S2A and B, ESI†). It was supposed after the phosphate group cleaved from the ALP enzyme. The l-tyrosine phosphate was replaced with l-tyrosine. Its self-assembled morphology was analyzed *via* TEM. Similar fibers with a diameter of 10 nm were observed (Fig. S3, ESI†), confirming that ALP transforms the l-Mito-FFYp micelles into l-Mito-FFY fibers.

### Cell cytotoxicity and cancer cell death mechanism

**Fig. 2:**
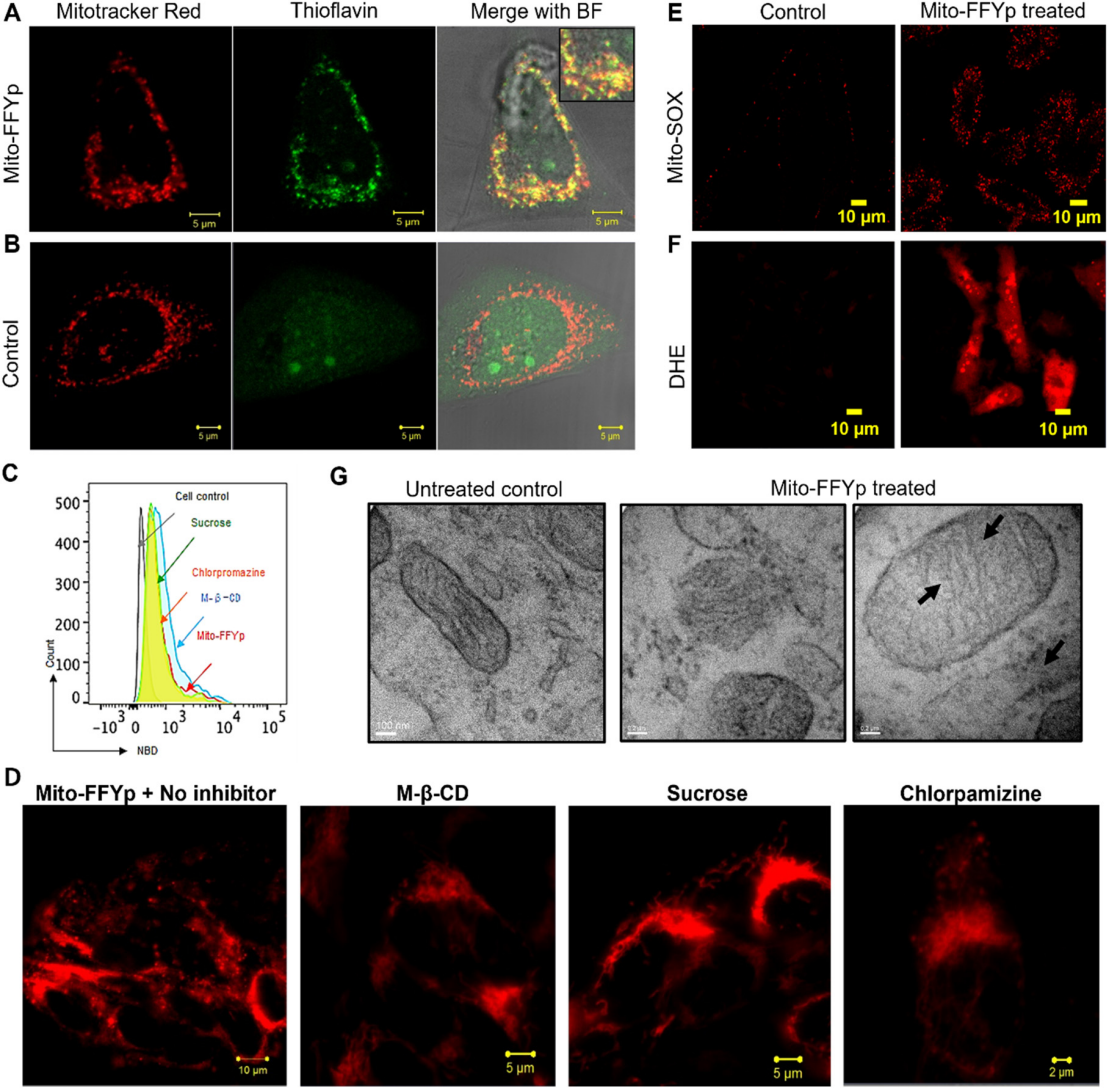
Confocal images showing (A) green fluorescence from thioflavin after treating the cells with l-Mito-FFYp and (B) the absence of green fluorescence without l-Mito-FFYp. (C) Fluorescence-activated cell sorting (FACS) analysis showing the cellular uptake of l-Mito-FFYp-NBD in the presence of endocytosis inhibitors. (D) Mitochondrial dysfunction induced by l-Mito-FFYp in the presence and the absence of endocytosis inhibitor. (E and F) The red fluorescence which represents ROS was observed when l-Mito-FFYp treated into the Saos-2 cell. (G) TEM images of SaOS-2 mitochondria with no treatment with l-Mito-FFYp and after treatment with l-Mito-FFYp showing the mitochondrial damage and fiber assembly inside.

Next, the cellular internalization and colocalization of l-Mito-FFYp in the Saos-2 cell lines (ALP overexpressed cell line) was analyzed after 24 h of incubation. A confocal microscopic analysis showed an overlap of the blue fluorescence of l-Mito-FFYp and the red fluorescence of Mitotracker red FM, suggesting that l-Mito-FFYp internalizes and colocalizes inside the mitochondria (Fig. S4, ESI†). To confirm the intramitochondrial fiber formation of l-Mito-FFYp, cell lines treated with l-Mito-FFYp were incubated with thioflavin (ThT), which is known to intercalate with β-fibers producing bright green fluorescence. The corresponding confocal imaging analysis showed a good overlap between the green fluorescence of l-Mito-FFYp and the red fluorescence of Mitotracker red FM, indicating that l-Mito-FFYp not only enters the mitochondria but also forms fibers (Fig. 2A). In contrast, no green fluorescence from the mitochondria was observed in the untreated control (Fig. 2B). To determine whether the *in situ*-generated l-Mito-FFYp undergoes self-assembly into nanofibers within the cell membrane or internalizes in the cell prior to the self-assembly, fluorescence-activated cell sorting (FACS) analysis was implemented with fluorophore conjugated l-Mito-FFYp. Since the blue fluorescence from pyrene butyric acid could not be clearly measured due to the lack of a proper filter. Therefore, we conjugated nitrobenzoxadiazole (NBD) dye^33,34^ to synthesize another peptide, l-Mito-FFYp-NBD (Fig. S5A, ESI†) to quantitatively confirm the cellular internalization of l-Mito-FFYp in the presence of endocytosis inhibitors. l-Mito-FFYp-NBD was synthesized by conjugating NBD-Cl to the secondary amine of the lysine side chain. Like l-Mito-FFYp, l-Mito-FFYp-NBD self-assembled into micelles under physiological conditions (Fig. S5B, ESI†). The absorption spectra of Mito-FF-NBD showed the absorption for pyrene (at 343 nm) and NBD (at 495 nm), confirming the successful incorporation of the NBD dye molecule into the l-Mito-FFYp structure (Fig. S5C, ESI†). The excitation of l-Mito-FFYp-NBD at 495 nm produced an emission peak at around 600 nm due to the NBD fragment, which enabled the FACS analysis. A flow cytometric analysis of Saos-2 cell lines treated with l-Mito-FFYp-NBD in the presence and absence of endocytosis inhibitors showed that the molecules were successfully internalized in the cells irrespective of the presence of endocytosis inhibitors (Fig. 2C). The Mitotracker red FM showed severe leakage from the mitochondria to the cytoplasm compared with the untreated control, which indicates that l-Mito-FFYp effectively induced mitochondrial fragmentation (Fig. S6A, ESI†). The same experiment was repeated in the presence of chlorpromazine, sucrose, and M-β-CD, finding that these endocytosis inhibitors did not restrict the mitochondrial dysfunction (Fig. 2D and Fig. S7, ESI†). Further, cellular uptake at 4 °C showed similar uptake at 37 °C. It suggests the l-Mito-FFYp peptides internalized to the cell through energy independent pathway. And treatment of the mitochondrial membrane potential indicator dye tetramethyl-rhodamine methyl ester (TMRM) with l-Mito-FFYp led to disappearance of the red fluorescence from the mitochondria both in the absence and presence of an endocytosis inhibitor, which confirmed the loss of mitochondrial integrity (Fig. S6B and S8, ESI†). Generally, intramitochondrial self-assembling peptides cause overproduction of reactive oxygen species (ROS)^35,36^ within the mitochondria as well as in the cytoplasm. To investigate whether l-Mito-FFYp induces overproduction of ROS within the mitochondria, l-Mito-FFYp-treated Saos-2 cells were treated with Mito-SOX dye, which is an indicator for mitochondrial ROS. In contrast with the untreated control, the l-Mito-FFYp-treated cells emitted red fluorescence, which was further confirmed by FACS (Fig. 2E and Fig. S9A, ESI†). In addition, staining with dihydroethidium (DHE) produced a deep red fluorescence from the l-Mito-FFYp treated Saos-2 cell line, as shown in confocal microscopy and flow cytometric analyses, suggesting that l-Mito-FFYp caused overproduction of ROS in the cell (Fig. 2F and Fig. S9B, ESI†). The cell TEM images showed undamaged mitochondria for the untreated control, whereas mitochondrial damage was observed after treatment with l-Mito-FFYp. (Fig. 2G) Moreover, the presence of fibers inside the mitochondria confirmed that the mitochondrial damage was induced by the intramitochondrial fibers.

**Fig. 3:**
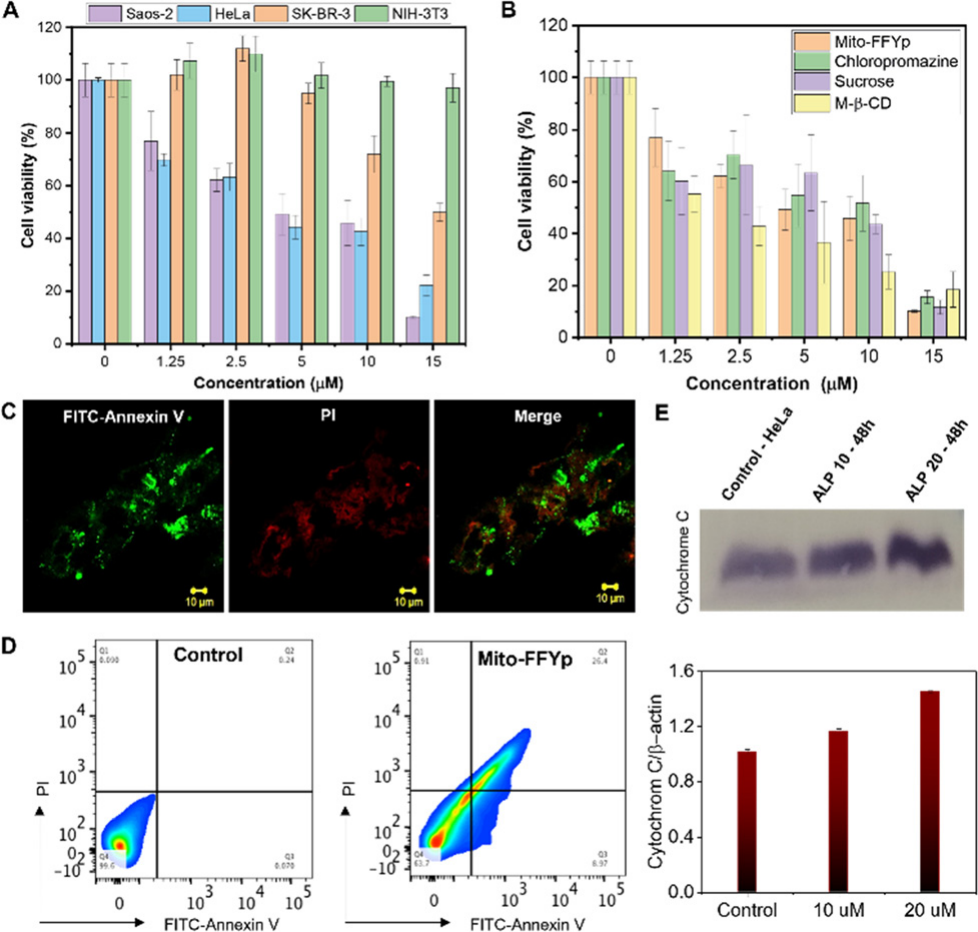
(A) Cell viability analysis of l-Mito-FFYp in Saos-2, HeLa, SK-BR-3 and NIH 3T3 cell lines for an incubation time of 48 h. (B) Cell viability analysis of l-Mito-FFYp in the presence of different endocytosis inhibitos. (C) Cell death induced by l-Mito-FFYp monitored after staining with FITC-Annexin/PI showing late apoptosis. (D) Flow cytometric analysis showing that the cell underwent late apoptosis after treatment with l-Mito-FFYp. (E) Western blotting analysis showing the release of cytochrome *C* after treatment with l-Mito-FFYp.

To confirm that the cytotoxicity of l-Mito-FFYp stems from its selectivity toward ALP and the consequent morphology transformation, the cytotoxicity was evaluated toward Saos-2, HeLa, SK-BR-3, and NIH-3T3 cell lines. SK-BR-3 and NIH-3T3 cell lines are known to express fewer ALP enzymes. The SK-BR-3 and NIH 3T3 cell lines showed almost 90% viability at a concentration of 10 μM. In contrast, 50% viability was observed for Saos-2 cells and HeLa cells at about 5 μM due to the high level of ALP expression.^37,38^ This suggests that the cytotoxicity of l-Mito-FFYp is selective toward the ALP enzyme expression (Fig. 3A). And the cytotoxicity of l-Mito-FFYp in the Saos-2 cell line was analyzed in the presence and absence of endocytosis inhibitors such as chlorpromazine (clathrin), sucrose (macropinocytosis), and M-β-cyclodextrin (caveolin). In both cases, the cell cytotoxicity analysis of l-Mito-FFYp by MTT assay showed a similar trend in toxicity with an IC_50_ value of ∼10 μM after 48 h of incubation (Fig. 3B). This result suggests that the endocytosis inhibitors cannot prevent the cellular internalization of l-Mito-FFYp, which in turn indicates that l-Mito-FFY diffuses freely inside the cell instead of internalizing as l-Mito-FFY fibers. These results are consistent with Fig. 2C and D. Fluorescein isothiocyanate (FITC)-annexin V/Propidium Iodide (PI) assays were conducted to determine the mechanism of cell death induced after treat with l-Mito-FFYp. Both red and green fluorescence was observed in the confocal imaging and FACS upon staining with FITC-annexin V/PI, which suggested that the cells underwent a late apoptosis after treatment with l-Mito-FFYp (Fig. 3C and D). These results indicate that Saos-2 cells treated with l-Mito-FFYp underwent apoptosis induced by mitochondrial membrane disruption. To further verify the important role of the phosphate moiety, we synthesized l-Mito-FFY compound (Fig. S2, ESI†). l-Mito-FFY showed high accumulation into the mitochondria (Fig. S10, ESI†) and induced damage to the mitochondria (Fig. S11, ESI†). However, l-Mito-FFY compound made the loss of mitochondrial integrity on healthy NIH-3T3 cell (Fig. S12, ESI†), and its cytotoxicity against NIH-3T3 was highly increased after 48 h incubation (Fig. S13, ESI†). When l-Mito-FFYp was treated with Nitro-blue tetrazolium chloride/5-bromo-4-chloro-3’-indolyphosphate p-toluidine salt (NBT-BCIP) solution, known as an ALP inhibitor, we observed that the toxicity was remarkably reduced, proving that the enzyme conversion process is essential (Fig. S14, ESI†). Altogether, these results indicate that the phosphate group grants more selectivity through EIMT process against cancer. A western blotting analysis showed that cytochrome *C* was released from the mitochondria after treatment with l-Mito-FFYp, indicating that the cell underwent mitochondrion-mediated apoptosis (Fig. 3E). Taken together, these results demonstrate the apoptosis of osteosarcoma cells induced by the EIMT of l-Mito-FFYp. After phosphoric acid was cleaved by the ALP enzyme overexpressed in the Saos-2 cell membrane, Mito-FFY was accumulated in the mitochondria. As a result, apoptosis occurred due to mitochondrial membrane disruption induced by self-assembled fibers inside the mitochondria.

## Conclusions

This work demonstrated the selective death of osteosarcoma cells as a result of the enzyme-instructed morphology transformation of drug-free agents. To prevent binding to negatively charged biomolecules, the substrate of ALP, phosphoric acid, was conjugated on tyrosine and added to the main backbone of the peptide. The synthesized l-Mito-FFYp formed micelles with a CAC value of 12 μM and a surface zeta potential of −25.6 mV. Upon reaction with the ALP enzyme, the l-Mito-FFYp micelles were converted into fibers and the surface potential changed to +33.5 mV. l-Mito-FFY formed fibers within the mitochondria in cancer cells, and this morphological transformation disrupted the mitochondrial membrane. In this process, the toxicity of the peptide increased with the expression level of the enzyme, with the Saos-2 cell line being the most affected. After enzymatic cleavage, the internalization in the cancer cells did not proceed *via* endocytosis but passive diffusion of the cleaved monomer l-Mito-FFY. Finally, the disrupted mitochondria from l-Mito-FFY released apoptotic protein cytochrome *C* into the cell cytoplasm, causing apoptosis. This strategy is anticipated to have clinical potential applications for anticancer treatment. l-Mito-FFYp micelle has stability on our body fluid compared to peptide monomer. It has an advantage after intravenous injection. Therefore, this strategy will be a tremendous effect on the osteosarcoma tumor composed of cancer cells with high ALP expression levels.

## Author contributions

J. H. R designed the work. J. H. R., M. T. J and S. J. directed the experiments. M. T. J and, S. J synthesized ALP enzyme responsive compound and characterized the compounds studied the cleavage process. And performed cell viability, confocal experiments for mitochondrial accumulation and mitochondrial dysfunction. B. J. performed flow cytometric analysis and western blot. The manuscript was written by M. T. J., S. J. and J. H. R.

## Conflicts of interest

The authors declare no competing financial interests.

## Supplementary Material

CB-003-D2CB00166G-s001
