# Supplementary material for: Enzyme-instructed morphology transformation of mitochondria-targeting peptide for the selective eradication of osteosarcoma
Source: RSC Chem Biol. 2022 Oct 17;3(12):1416–21. doi: 10.1039/d2cb00166g (PMC9709777; doi:10.1039/d2cb00166g)
Supplement: CB-003-D2CB00166G-s001 [file CB-003-D2CB00166G-s001.pdf]

Electronic Supplementary Material (ESI) for RSC Chemical Biology.
This journal is © The Royal Society of Chemistry 2022

Supporting Information

Enzyme instructed morphology transformation of mitochondria targeted drug-free agents in the cell membrane for the selective eradication of osteosarcoma.

M. T. Jeena,‡[a] Seongeon Jin,‡[a] Batakrishna Jana,[a] and Ja-Hyoung Ryu,*[a]

Materials. All peptides were synthesized manually on a 0.25 μM scale. The products were purified by high-performance liquid chromatography (HPLC, Agilent Technologies, USA) with a C-18 reverse column in ACN/water mixture. Amino acids were purchased from Bead Tech, Korea and APEX Bio, Houston, USA. Mito-SOX, Mitotracker Red FM, DHE were purchased from Thermo Fisher Scientific, Korea. The tetramethyl rhodamine methyl ester (TMRM) was purchased from Santa Cruz Biotechnologies, Korea. The successful synthesis of peptides was confirmed with matrix-assisted laser desorption/ionization (MALDI-TOF/TOF, Ultraflex III). The confocal laser scanning microscopy images were taken with LSM 780.The TEM images were obtained using a BioTEM system (JEM 1400).

Synthesis of peptide. The peptide Mito-FFYp were synthesized by the standard 9-fluorenylmethoxycarbonyl(Fmoc) solid-phase peptide synthesis on a 0.25 μM scale. The synthesized FFYp peptides were treated with 1-pyrene carboxylic acid (500 μmol) and O- (benzotriazol-1-yl)-N,N,N′,N′-tetramethyluronium hexafluorophosphate (HBTU) (500 μmol) in the presence of diisopropyl ethyl amine (DIPEA, 500 μmol) and allowed to stir at room temperature for 24 h in DMF. The resin was collected by filtration and washed with dimethylformamide (DMF) to remove unreacted chemicals. The product was cleaved from the resin with cleavage cocktail (TFA/ Water/Tri isopropyl amine mixture (9.5:0.5:0.5)), and products were precipitated in cold ether, purified by HPLC, and confirmed by mass analysis using MALDI-TOF/TOF. To achieve the triphenyl phosphonium (TPP) conjugation, synthesized peptide (0.02 mmol) was treated with 1-hexyl triphenylphosphonium bromide salt (0.04 mmol) with triethyl amine (0.02 mmol) in DMF and allowed to stir for 12 h at room temperature. The pure product was collected using HPLC, freeze-dried, and used for further studies. The peptide was obtained with 90 % yield.

Cell culture. Cell lines were obtained from ATCC and cultured in DMEM, L-15, or RPMI (Life Technologies) containing 10% fetal bovine serum (FBS; Life Technologies) and 1% penicillin/streptomycin (Life Technologies) at 37 °C in a humidified atmosphere of 10% CO_2. Cell viability was measured using MTT assay.

Imaging mitochondrial dysfunction. Saos-2 cells were seeded on Lab Teck II slide chamber at 80% confluence in DMEM (Life Technologies) supplemented with 10% FBS and 1% penicillin/streptomycin and incubated at 37 °C under 5% CO2. After 24h, cells are incubated with 20 μM of Mito-FFYp for another 6h. Saos-2 Cells were labelled with Mito Tracker Red FM, Mito-SOX Red, DHE and TMRM to observe mitochondrial fragmentation, mitochondrial ROS generation, cellular ROS generation and

mitochondrial depolarization respectively.

Imaging of apoptosis/necrosis-dependent cell death. HeLa cells were seeded on a Lab Tek II slide chamber at 80% confluence in DMEM (Life Technologies) supplemented with 10% FBS and 1% penicillin/streptomycin at 37 °C under 5% CO2. The cells were then incubated with the Mito-FFYp at 20 μM for 24 h. The cell culture medium was then replaced with Alexa Fluor 488-conjugated annexin V and PI in 1 ml of annexin binding buffer and incubated for 15 min at 37 °C. The cells were then washed gently, the medium was replaced with DMEM medium, and the cells were analyzed using an LSM780 laser confocal scanning microscope.

Flow cytometry analysis. HeLa cells were seeded in a 25-mL T-flask (Thermo Scientific) in DMEM (Life Technologies) supplemented with 10% FBS and 1% penicillin/streptomycin at 37 °C under 5% CO_2. The cells were then incubated with the peptides at 20 μM of Mito-SA for a period of 12 h. The cells were stained with Mio-SOX, DHE and TMRM as per the recommended time period for each case. The cells were washed, trypsinized and collected to analyses by the flow cytometer.

Transmission electron microscopic analysis of peptides. A drop of 100 μM deionized water solution of peptides placed on a Formvar/carbon-coated copper grid and allowed to evaporate under ambient conditions. The sample was stained with 2 wt % uranyl acetate solution and allowed to evaporate for 5 min, and excess solution was removed with a filter paper. The specimen was observed with JEM-1400 TEM operating at 120 kV.

Cell TEM sample preparation protocol. Saos-2 cells were grown in a 24-well plate on 15 mm diameter Theramanox coverslips (Nalge Nunc International, NY) in 1 mL serum containing media with 80% confluency. The cells were treated with 20 μM of Mito-FFYp for 12 h. The cells were then fixed using 4% paraformaldehyde for 30 min at room temperature and then washed with 0.1 M Na-PO4 buffer + 5% sucrose (1 mL), 3 times over 30 min at room temperature. The TEM samples of the cell were prepared according to the reported protocol

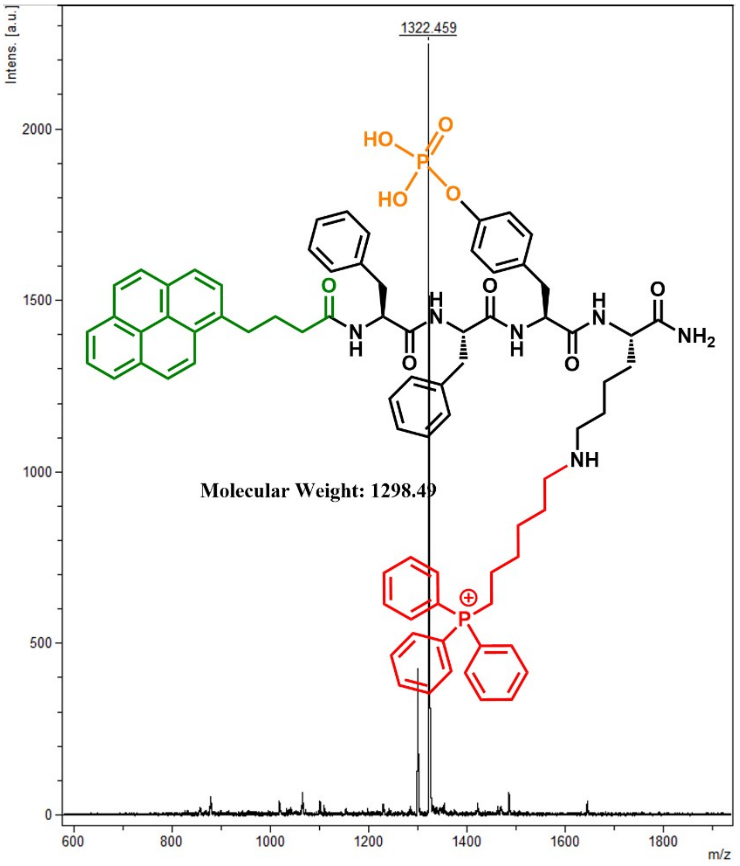

Figure S1. Chemical structure of Mito-FFYp and MALDI-TOF spectra of Mito-FFYp after HPLC purification that confirms the successful formation of the product.

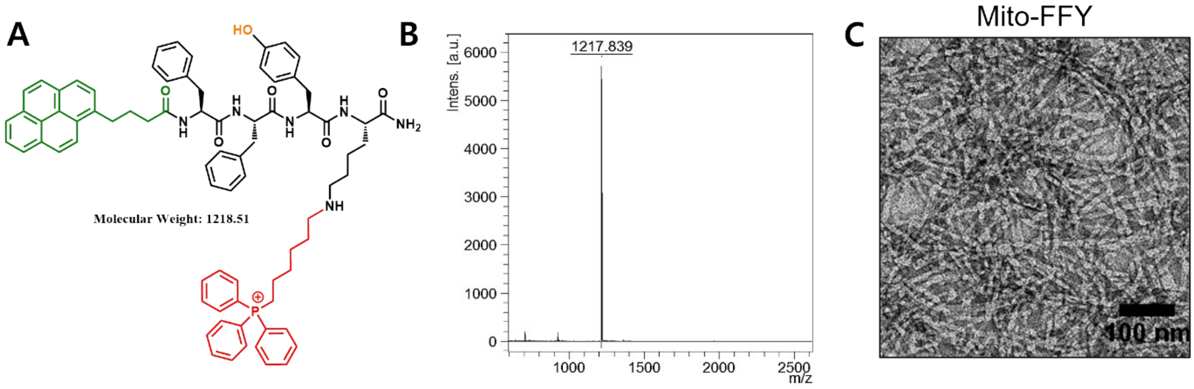

Figure S2. A) Chemical structure of Mito-FFY, B) MALDI-TOF spectra of Mito-FFY after HPLC purification that confirms the successful formation of the product, C) TEM images of Mito-FFY showing the presence of nanofibers of diameter ~ 10 nm.

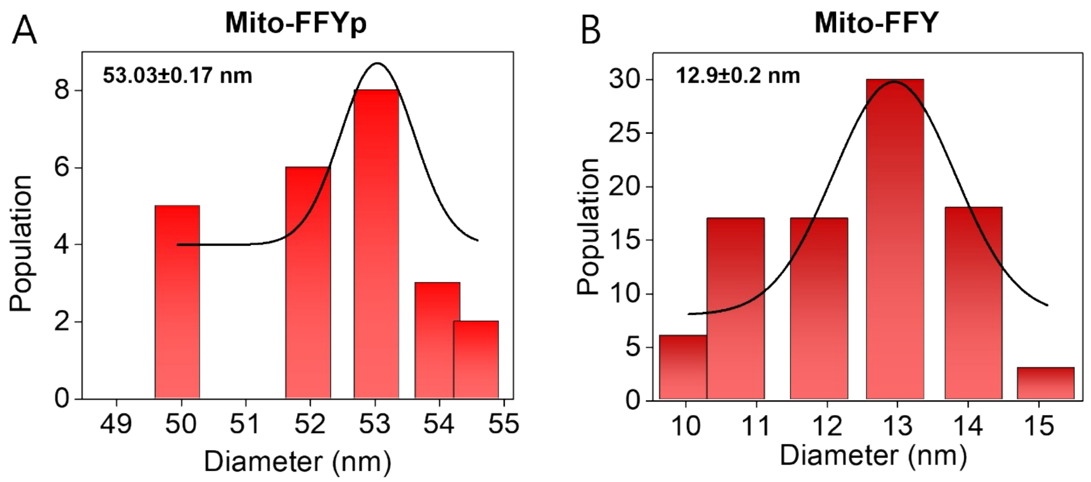

Figure S3. Histograms of the diameter distribution of the micelle of Mito-FFYp and fibers of Mito-FFY.

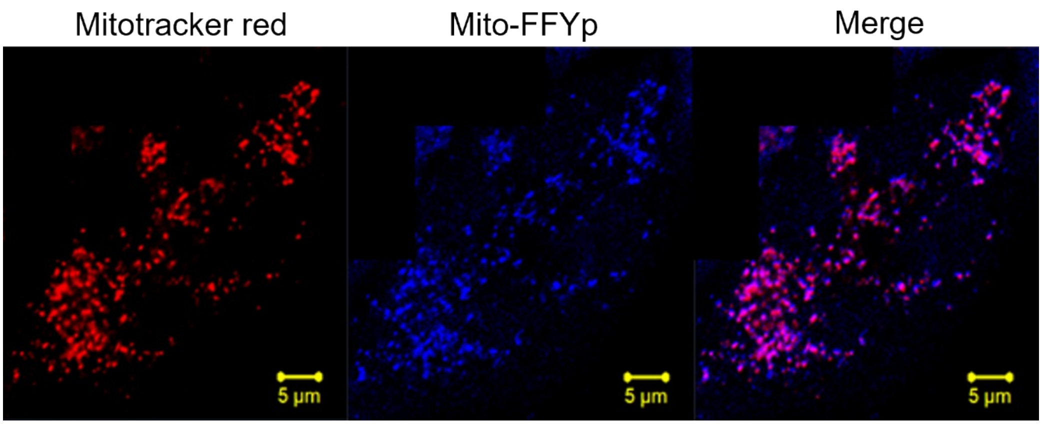

Figure S4. Cellular internalization of Mito-FFYp in the SaOS-2 cell lines showing their co-localization inside the mitochondria.

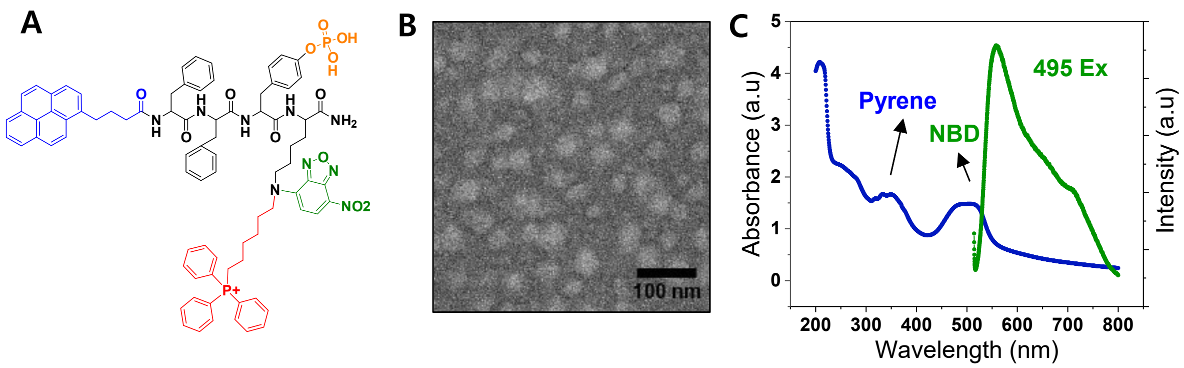

Figure S5. A) Chemical structure of Mito-FFYp-NBD, B) TEM images of Mito-FFY showing the presence of nanofibers of diameter ~ 10 nm. C) UV-vis absorption spectra and fluorescence emission spectra of Mito-FFYp-NBD

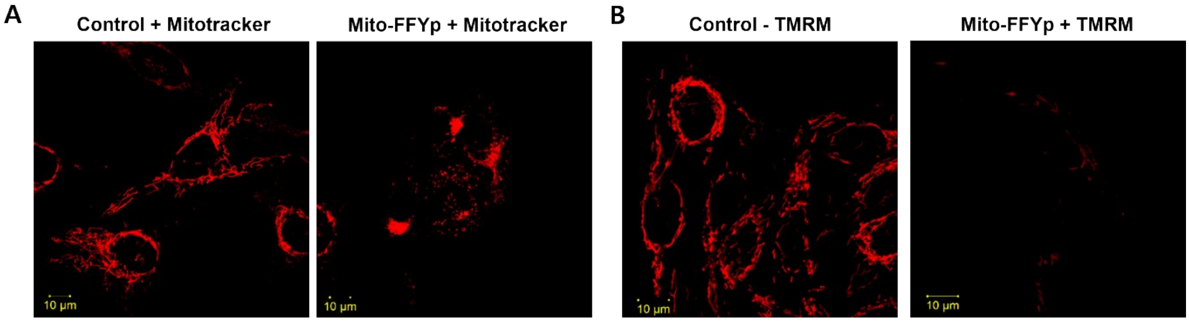

Figure S6. Mitochondrial dysfunction induced by Mito-FFYp which is measured by labeling with A) Mitotracker, B)TMRM.

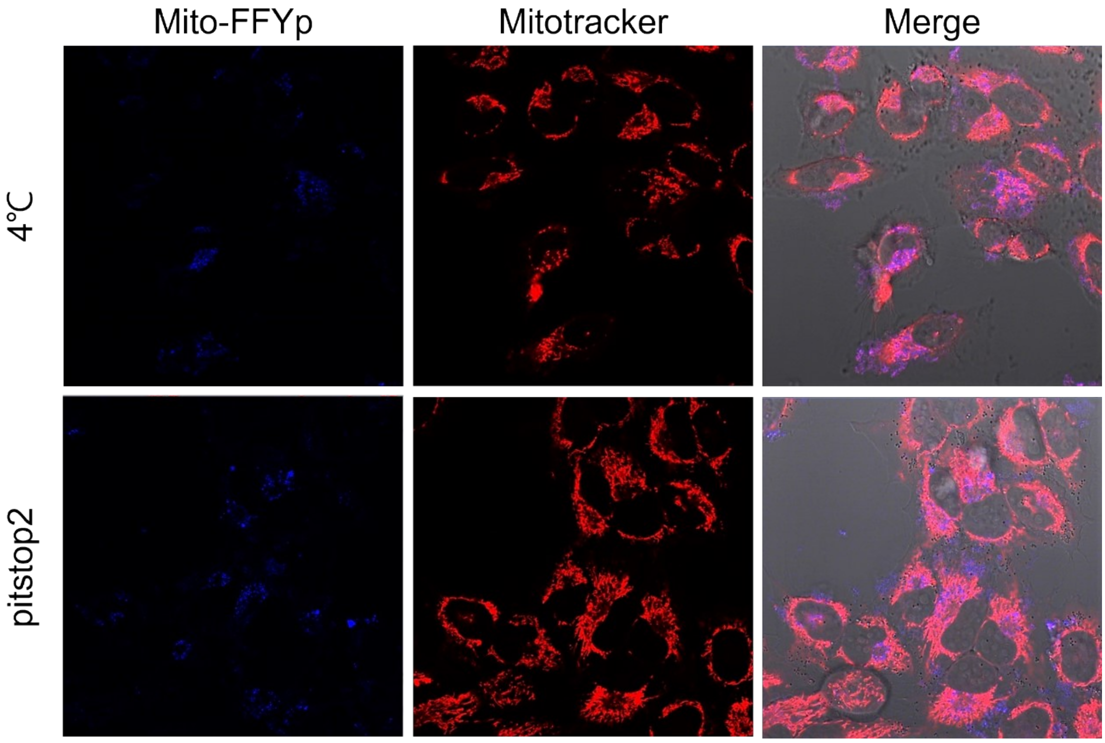

Figure S7. Confocal images showing cellular uptake at 4 ℃ condition and in presence of the clathrin-mediated endocytosis inhibitor, pitstop2.

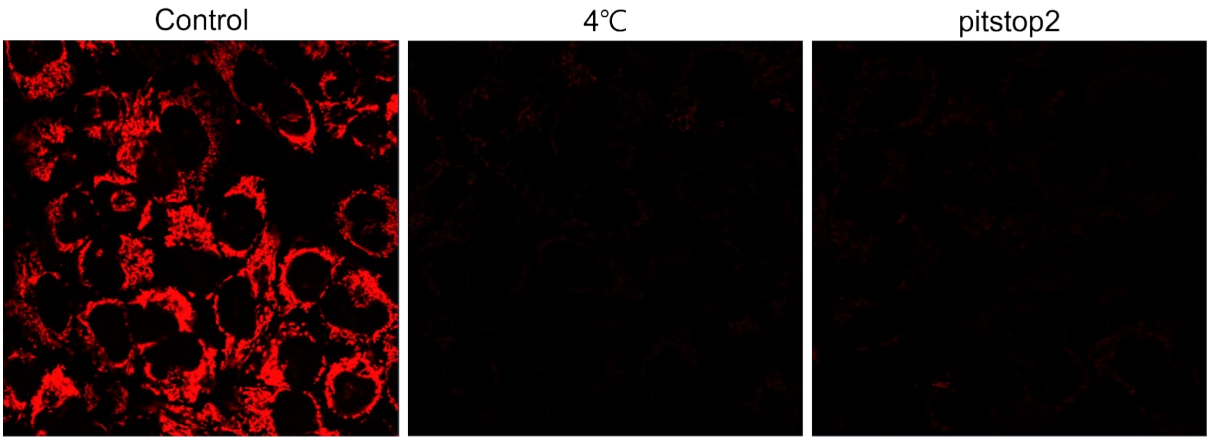

Figure S8. Mitochondrial dysfunction at Saos-2 cell induced by L-Mito-FFYp which is measured by labeling with TMRM. L-Mito-FFYp compound was treated at 4℃ or co-incubated with pitstop2.

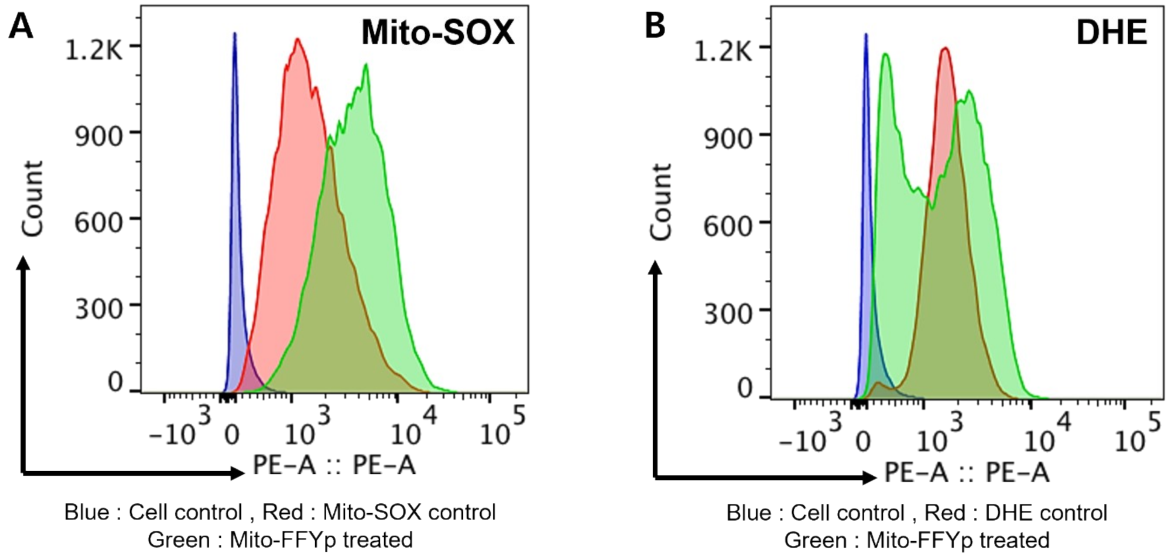

Figure S9. Corresponding flow cytometric analysis of Figure 2E, F.

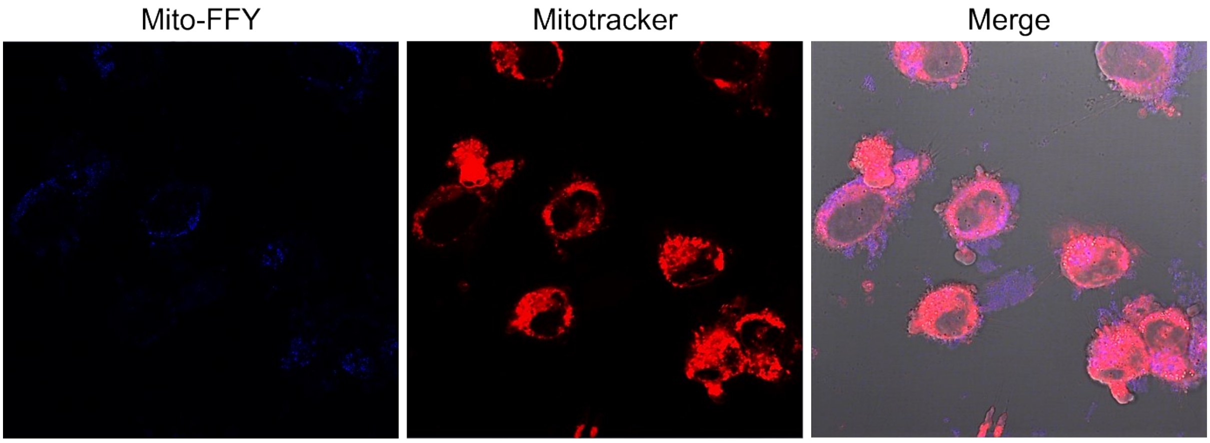

Figure S10. Confocal images showing cellular uptake of Mito-FFY at Saos-2 cell.

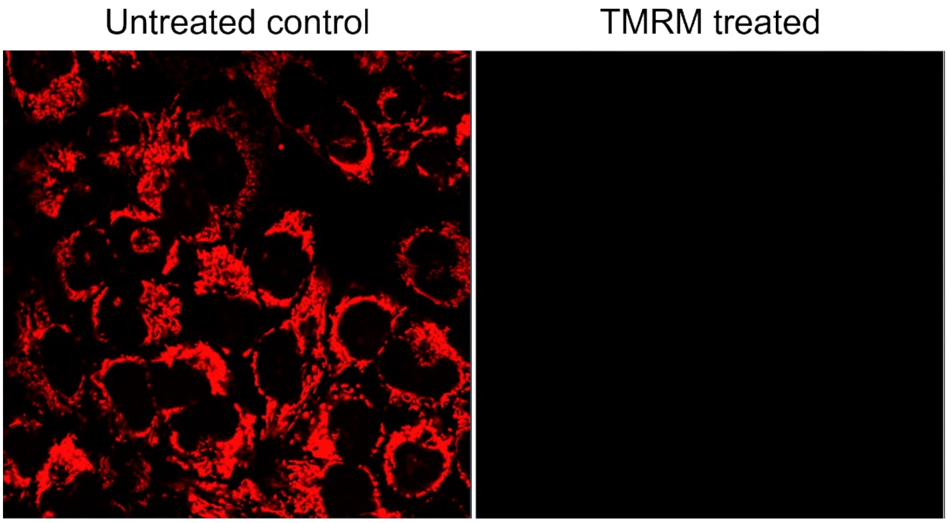

Figure S11. Mitochondrial dysfunction at Saos-2 cell induced by Mito-FFY which is measured by labeling with TMRM.

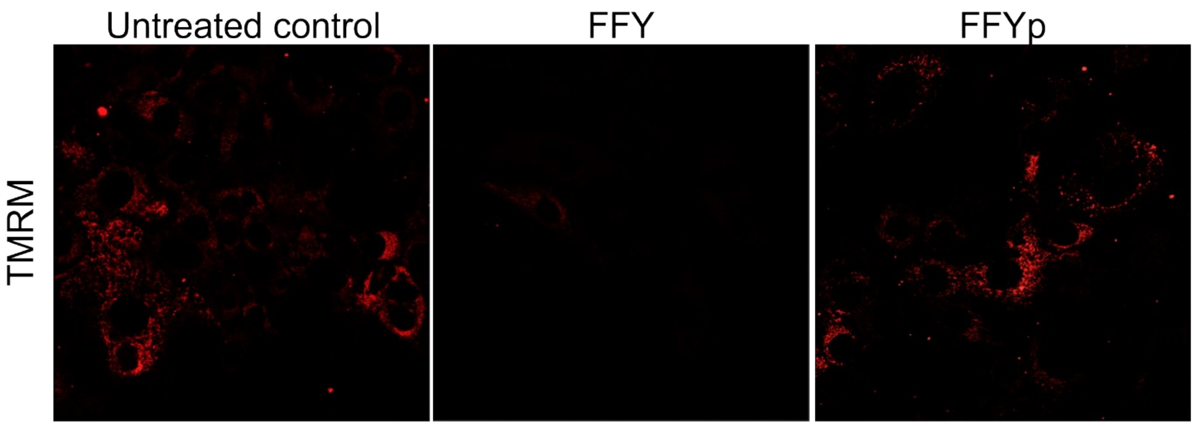

Figure S12. Mitochondrial dysfunction at NIH-3T3 cell induced by Mito-FFY which is measured by labeling with TMRM.

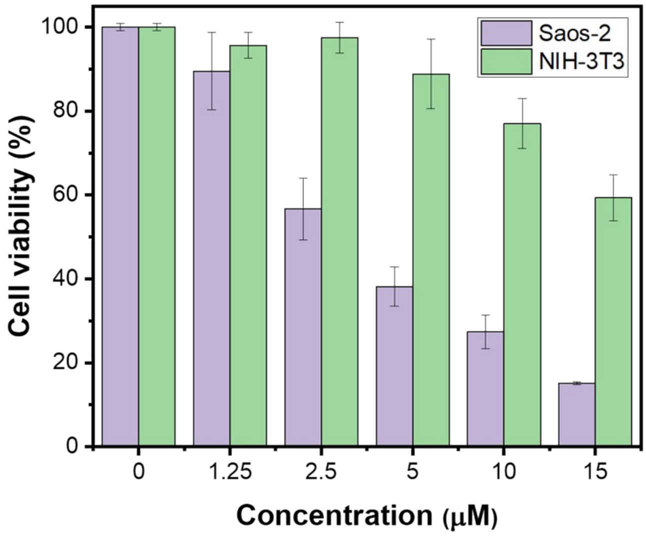

Figure S13. Cell viability analysis of Mito-FFY in Saos-2 and NIH 3T3 cell lines for an incubation time of 48 h.

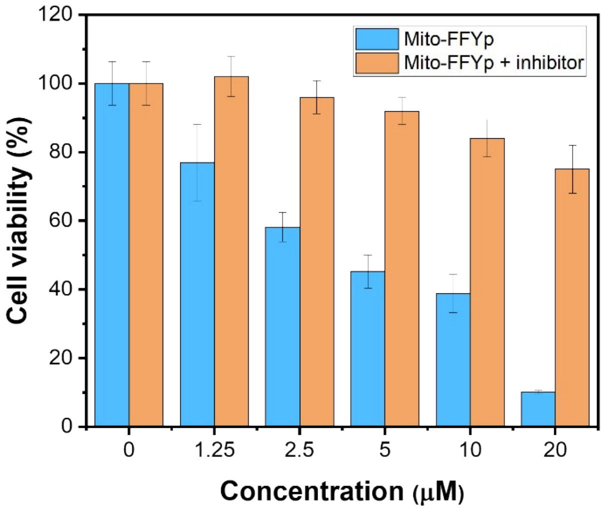

Figure S14. Cell viability analysis of Mito-FFYp in the presence of ALP inhibitor, NBT-BCIP.
